# The Crosstalk between Myeloid Derived Suppressor Cells and Immune Cells: To Establish Immune Tolerance in Transplantation

**DOI:** 10.1155/2016/4986797

**Published:** 2016-10-27

**Authors:** Chao Zhang, Shuo Wang, Cheng Yang, Ruiming Rong

**Affiliations:** ^1^Department of Urology, Zhongshan Hospital, Fudan University, Shanghai, China; ^2^Shanghai Key Laboratory of Organ Transplantation, Shanghai, China; ^3^Department of Plastic Surgery, Zhongshan Hospital, Fudan University, Shanghai, China; ^4^Department of Transfusion, Zhongshan Hospital, Fudan University, Shanghai, China

## Abstract

Myeloid derived suppressor cells (MDSCs) are a heterogeneous population of myeloid precursor and progenitor cells and endowed with a robust immunosuppressive activity in multiple pathophysiological conditions. Recent studies have uncovered the crosstalk between MDSCs and immune cells (i.e., natural killer cells, dendritic cells, macrophages, natural killer T cells, and regulatory T cells) and its role in the establishment and maintenance of immune tolerant microenvironment in transplantation. Considering their strong immunosuppressive capability, MDSCs could become a prospective clinical regimen during transplantation tolerance induction, resulting in long-term graft survival with decreased or without immunosuppressive drugs. The review summarized recent research advances in this field and looked ahead at the research directions in the future.

## 1. Introduction

One of the major concerned questions in solid organ transplantation is how to establish long-term allograft survival that is free from immunosuppressive strategies. The most promising answer to this question is to establish immune tolerance in the recipient. Decades of years have witnessed the attempts to achieve this goal from cotransplantation with hematopoietic stem cell to the induction of chimerism. Recently, emerging evidence highlights that myeloid derived suppressor cells (MDSCs) have great potential as a novel immune intervention for inducing transplant tolerance.

MDSCs are a heterogeneous population of cells composed of progenitors and precursors of myeloid cells such as dendritic cells, macrophages, and granulocytes at various stages of differentiation [1, 2]. In mice, MDSCs are generally identified by coexpression of surface markers CD11b and Gr-1, but with two subtypes, G-MDSCs and M-MDSCs, based on their distinct expression of Ly-6C and Ly-6G [3]. However, human MDSCs cannot be uniformly identified by specific markers so far. Some investigators defined human MDSCs as CD11b+CD33+HLA-DRlow/− cells [4], but without consensus in academics. Bartmann et al. affirmed in their study that human MDSCs could also be subdivided into two main subsets: CD15+CD14−CD11b+CD33+HLA-DRlow/− G-MDSCs and CD15−CD14+CD11b+CD33+HLA-DRlow/− M-MDSCs [4]. The reason why these cells with different origins can be summarized as one group is that they share two common characteristics: one is that they are all staying in an immature state; the other is that they are able to exert strong suppressive activity on T cell proliferation and activation. In terms of the mechanism involved in T cell inhibition, G-MDSC subtype is dependent on reactive oxygen system (ROS) while M-MDSC subtype is through high expression of inducible nitric oxide synthase (iNOS) and nitric oxide (NO) [5, 6]. High expression of arginase-1 (Arg-1) is of pivotal importance for both of these two subtypes [7]. MDSCs were originally reported in tumor-associated animal models [8]. Locating in the tumor microenvironment, MDSCs contribute to tumor growth and metastasis via suppressing tumor antigen-driven activation of T cells [9]. MDSCs have also been shown to produce vascular endothelial cell growth factor (VEGF), *β*-fibroblast growth factor (*β*-FGF), VEGF analogue Bv8, and matrix metalloproteinase 9 (MMP9), all of which are essential for angiogenesis and tissue invasion at tumor sites [10]. Interestingly, MDSC's role of promoting tumor progression reminds us that they can serve as a potential immunotolerant inducer in transplant immunity. Recent studies demonstrated that MDSCs interacted with multiple immune cells and contributed greatly to the induction of immune tolerance in organ or cell transplants. This review summarized the crosstalk between MDSCs and natural killer cells, dendritic cells, macrophages, natural killer T cells, and regulatory T cells as well as the effect on kidney transplant, skin transplant, pancreatic islet transplant, cardiac transplant, and graft-versus-host disease in hematopoietic stem cell transplantation.

## 2. The Crosstalk between MDSCs and Immune Cells

### 2.1. MDSC and Natural Killer (NK) Cell

Currently, there still exists controversy about the influence of MDSC on NK cells. However, most investigators agreed that the coculture with MDSC impaired NK cell's recognition and cytotoxic effects on alloantigens, leading to immune tolerance in transplants. The study showed that MDSC was able to downregulate the expression of CD247 on the surface of NK cells, which was the key subunit of natural cytotoxicity receptor (NCR) NKp46, NKp30, and Fc*γ* RIII. The downexpression of CD247 inhibited the development and cytotoxic activity of NK cells, therefore attenuating its killing effect on allogenic antigens [11]. Besides, the expression of NKG2D, a killer lectin-like receptor (KLR) which could initiate killing effects of NK cells, and the secretion of interferon- (IFN-) *γ* were also downregulated after coculture [12]. Interestingly, the inhibition of NK cell activity by MDSC was reversed when membrane-bound transforming growth factor- (TGF-) *β* expressed on MDSCs was blocked, which indicated that the inhibitory effect was dependent on cell-cell contact [13].

### 2.2. MDSC and Dendritic Cell (DC)

Most investigations on the interaction between MDSCs and DCs were implemented on animal models or patients with tumors. These investigations reported that MDSCs could inhibit DCs maturation in tumor microenvironment and prevent them from differentiation, thereby inducing immune tolerance to tumor-specific antigens [14]. The main mechanism in this process was that vascular endothelial growth factor (VEGF) and interleukin- (IL-) 10 in tumor microenvironment downregulated the expression of major histocompatibility complex (MHC) II and costimulators on DCs by activating signal transducer and activator of transcription (STAT) 3 signaling [15, 16]. Another research on the MDSCs isolated from the patients with melanoma revealed a different mechanism involving MDSC-mediated retardant maturation of DC: MDSCs could interfere with the process of antigen capture and the migration of immature DC to secondary lymphoid organs, both of which are essential for DC maturation [17, 18]. In addition, MDSC was also reported to alter the cytokine profile secreted by DCs [19]. Despite the development regarding the crosstalk between MDSC and DC, the scientific academics have not illuminated whether MDSC suppresses the process of DC maturation directly or MDSC just redirects the differentiation of immature DCs. Besides, one fact that must be clarified is that seldom researches are implemented on animal transplant model or relevant clinical settings so far, which restricts our understanding in this field.

### 2.3. MDSC and Macrophage

Firstly, the crosstalk between MDSC and macrophages altered the cytokine secretion profiles of both: IL-10 secreted by MDSC decreased the expression of IL-6, IL-12, and tumor necrosis factor- (TNF-) *α* while it increased the expression of NO in macrophages. In return, IL-6 produced by macrophages could indirectly regulate IL-10 secretion by MDSC [20]. MDSC was demonstrated to participate in the phenotype switch from proinflammatory MI subtype to anti-inflammatory MII subtype, thereby rebalancing the immune response in transplant towards immune tolerance [21, 22]. Other studies concluded that the interaction of MDSC and macrophages were strengthened in inflammatory milieu [23]. For example, in the existing of proinflammatory cytokine IL-1*β* or proinflammatory lipid prostaglandin (PGE) 2, MDSC produced significantly greater amount of IL-10 [24, 25]. Secondly, after coculture with MDSC, the expression of MHC II molecules was downregulated, and thus macrophages were unable to present alloantigens as professional antigen-presenting cells, resulting in specific unresponsiveness of T cells to alloantigens [26, 27]. Interestingly, this process was dependent on the expression of IL-10, revealing the vital role of IL-10 in MDSC's function [27].

### 2.4. MDSC and Natural Killer T (NKT) Cell

NKT cells can be divided into two subtypes: type I NKT cells, also known as inducible NKT cells, participate in antitumor immunity, and on the contrary, type II NKT cells facilitate tumor progression [28, 29]. The investigations on the relationship between MDSC and NKT cells are limited. One study showed that IL-13 produced by type II NKT cells augmented the accumulation of MDSCs in target organs [30]. A recent research found that the crosstalk of MDSC and NKT cells contributes to immunotolerant microenvironment in a clinical regimen for immune tolerance induction [31]. In the microenvironment, NKT cells were activated to produce a great number of IL-4, which stimulated MDSC's expansion and activation [31].

### 2.5. MDSC and Regulatory T Cells (Tregs)

Being different from conventional T cells, Tregs are a group of T cells that exert immunoregulatory functions once activated by specific antigens [32]. The relationship between MDSCs and Tregs has been illustrated in abundant studies in vivo and in vitro. Actually, it is widely acknowledged that the induction of Tregs is one of the most important mechanisms involved in MDSC-mediated T cell inhibition [5, 33, 34]. The induction of Treg required both IFN-*γ* and IL-10 secreted by MDSC, indicating that the crosstalk was not dependent on cell-cell contact. The cytotoxic T-lymphocyte antigen (CTLA) 4 was also required for the injection of anti-CTLA-4 antibodies into tumor-bearing mice leading to blockade of tumor growth [35–37]. Based on the results regarding the effect of MDSC in transplant tolerance, we believe that the crosstalk between MDSC and Tregs is a priority in contributing to immunotolerant microenvironment.

In conclusion, the crosstalk between MDSCs and these immune cells not only results in the impaired capability of eliciting specific recognition and response to alloantigens, but also promotes the expansion and accumulation of immunoregulatory participators such as Tregs and MDSCs themselves, thereby contributing to the induction of immune tolerance in tumor sites or allografts.

## 3. Induction of Transplant Tolerance by MDSCs

### 3.1. Kidney Transplant

The study of Vanhove's group showed a significant accumulation of MDSCs in rat kidney transplantation [38]. In this study, MDSCs have a nonspecific immunosuppressive activity both in vivo and in vitro. The suppressive function was dependent on iNOS in isolated MDSC as well as in graft-infiltrating MDSC, and the administration of the iNOS inhibitor amino guanidine induced the rejection of accepted allografts [38, 39]. Surprisingly, CD4+CD25+Foxp3+ regulatory T cells were insensitive in vitro to MDSC-mediated suppression. These results presented the crosstalk between these two cell types in immune tolerance. More recently, clinical significance of MDSCs in renal transplantation with acute T cell-mediated rejection has been investigated. Allograft function was significantly increased in patients with high accumulation of MDSCs. Furthermore, isolated MDSCs from recipients are able to expand Treg cells and inhibit production of IL-17 in vitro [40]. Another clinical investigation found that elevated frequencies of circulating CD14− and CD14+ MDSCs were found in the recipients of renal transplants. Furthermore, CD14− MDSCs were found to be associated with higher occurrence of squamous cell carcinoma in these patients [41].

### 3.2. Skin Transplant

In a model of skin allograft in mice, the mechanism of MDSCs in transplant tolerance was demonstrated to involve the inhibitory receptors Ig-like transcript 2 (ILT2), an inhibitory T cell receptor (TCR) whose activation caused the inhibition of T cell activation. In this study, ILT2 interaction with human leukocyte antigen- (HLA-) G was shown to induce expansion of MDSCs with significant suppressive activity. In addition, survival of skin allografts was prolonged after adoptive transfer of MDSC from ILT2 transgenic mice and histologic evaluation of the allografts, showing that MDSCs from ILT-2 transgenic mice were recruited to the graft. ILT2 transgenic mice also have an increased expression of Arg1, most likely resulting from upregulated IL-4 and IL-13 in MDSCs [42]. Another study reported that adoptive transfer of MDSCs from lipopolysaccharide- (LPS-) treated mice in untreated recipients significantly prolonged skin allograft survival. In this study, they identified heme oxygenase-1 (HO-1), a stress-responsive enzyme of immunoregulatory properties, as the main mechanism by which MDSC regulated alloreactive T cells. Importantly, the fact that blockade of HO-1 before MDSC transfer prevented the delay of skin allograft rejection revealed a new immunosuppressive mechanism relevant for transplantation in addition to iNOS and arginase-1 [43]. In another skin graft model in mice, the in vivo induction of Gr-1+CD11b+ MDSC by Neupogen, the recombinant human granulocyte colony-stimulating factor (rhG-CSF), or the induction of CD4+Foxp3+ Treg by IL-2 complexes (IL-2C) similarly prolonged allograft survival [44]. Interestingly, when mice were treated with both IL-2C and Neupogen, a further increase of Tregs was recorded. This observation suggested a possible crosstalk between MDSC and Treg to prolong allograft survival.

### 3.3. Pancreatic Islet Transplant

Adoptive transfer of granulocyte-macrophage colony-stimulating factor (GM-CSF) +IL-6-inducing MDSCs from bone marrow cells could prevent allograft rejection and allow long-term survival of pancreatic islet allografts. This transplant tolerance was dependent on the expression of regulatory transcription factor CCAAT/enhancer binding protein beta (C/EBP*β*) in MDSCs, which suggested that C/EBP*β* may work as a critical regulator of the immunosuppressive environment [45]. Another group demonstrated that with injecting the mixture of cotransplantation of 2.5 × 10^6^ MDSCs and islet cells into diabetic mice, the survival of the islet cell allograft was significantly prolonged without requirement of immunosuppression. In this process, both in vitro and in vivo data presented that B7-H1 was absolutely indispensable for MDSC to exert immune tolerant activity [46]. This group later reported that iNOS played a key role in MDSC-mediated T cell unresponsiveness in islet cell transplant. iNOS^−/−^ MDSCs largely lost their ability to induce islet allograft tolerance [47]. This study held great potential of MDSC as a novel adjunctive immunotherapy for islet transplantation and may overcome the allograft rejection in islet cell transplants.

### 3.4. Cardiac Transplant

It is demonstrated that the number of CD11b+CD115+Gr1+ monocytic MDSCs was increased in a mouse model of heart transplantation. Shortly after transplantation, these MDSCs migrated from bone marrow where they generated to the allograft where they promoted the induction of Treg and prevented adaptive immune responses [34]. This result suggested that mobilization of bone marrow CD11b+CD115+Gr-1+ MDSCs under sterile inflammatory conditions could induce indefinite cardiac allograft survival. In another study, Luo's group demonstrated the expansion of two subpopulations of MDSCs induced by donor splenocytes treated with the chemical cross-linker ethylcarbodiimide (ECDI-SPs) was important for cardiac allograft protection [48]. Lastly, mammalian target of rapamycin (mTOR) inhibitors are the main immunosuppressive drugs for organ transplant recipients. The results from murine cardiac transplant model revealed that rapamycin treatment led to the recruitment of MDSCs and increased their expression of iNOS. Moreover, adoptive transcoronary arterial transfer of MDSCs from rapamycin-treated recipients prolonged allograft survival. The mTOR and Raf/MEK/extracellular signal regulated kinase (ERK) signaling pathways played an important role in MDSC expansion after rapamycin treatment [49].

### 3.5. Graft-versus-Host Disease (GVHD)

Allogeneic hematopoietic stem cell transplantation will initiate GVHD, but the effect of MDSCs on GVHD is not still fully understood. In the study of Zhou's et al., embryonic stem (ES) cells and bone marrow hematopoietic stem (HS) cells derived MDSCs were reported to exhibit strong suppression against T cell proliferation via multiple mechanisms involving iNOS and IL-10. They were also capable of inducing the development of CD4+CD25+Foxp3+ Tregs. Interestingly, adoptive transfer of ES-MDSCs can effectively prevent lethal GVHD in mice and lead to long-term survival among treated mice [50]. Highfill et al. demonstrated similar effect of bone marrow-derived MDSCs in preventing GVHD, however through a different mechanism involving Arg1 [51]. Wang et al. investigated the dynamic changes and effects of MDSCs in GVHD development and found that adding functional MDSCs in donor graft alleviated GVHD, whereas removal of MDSCs in vivo exacerbated GVHD. However, the occurrence of GVHD is not necessary for increase of MDSCs [52].

## 4. Final Remarks

**Figure 1:**
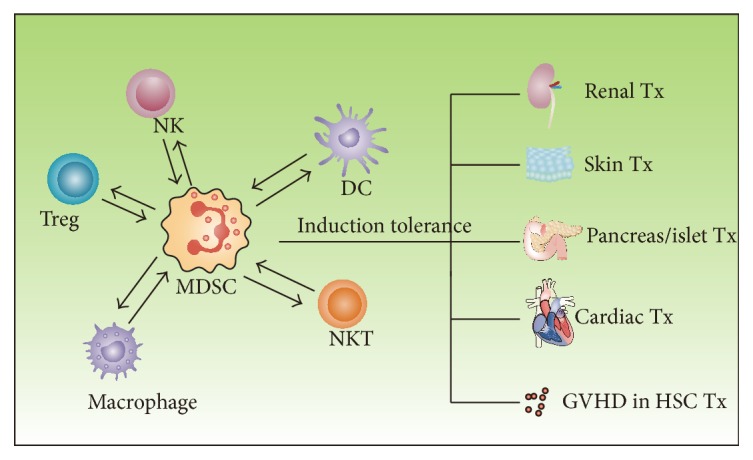
The role of MDSCs in immune tolerance induction of transplant. The crosstalk between MDSCs and immune cells such as NK cells, DCs, Tregs, macrophages, and NKT cells contributes to the establishment of immune tolerance in kidney transplant, skin transplant, pancreatic islet transplant, cardiac transplant, and GVHD in HSC transplant (NK cells: natural killer cells; DCs: dendritic cells; Tregs: regulatory T cells; NKT cells: natural killer T cells; GVHD: graft-versus-host disease; HSC: hematopoietic stem cell).

In this review, we summarized the crosstalk between MDSCs and natural killer cells, dendritic cells, macrophages, natural killer T cells, and regulatory T cells as well as the effect on kidney transplant, skin transplant, pancreatic islet transplant, cardiac transplant, and graft-versus-host disease in hematopoietic stem cell transplantation (Figure 1). MDSCs certainly have great function in attenuating or delaying graft rejection and in inducing allograft immune tolerance, which makes MDSCs a prospective strategy to control the ensuing graft rejection without weakening the whole immune system of the recipients. Despite the breakthroughs we have achieved in the mechanism of MDSC-mediated immune tolerance, greater efforts are still needed in the following respects. Firstly, cell-based regimens required a rather large amount of identified and purified cells to exert the expected therapeutic effects. Thus, clinically applicable protocols for expanding MDSCs ex vivo would provide a significant boost for translational application. Secondly, pharmacodynamics and pharmacokinetics assessments after MDSC administration are indispensable for determining whether or not these reagents could be used in clinic. How long is half-life period of MDSCs in vivo? Will MDSCs cause allergic reactions within the body as allergens? The safety of these heterogeneous cells should also be considered since severe adverse effects are not permitted regardless of the therapeutic effect. Last but not least, current studies on the crosstalk between MDSCs and immune cells are mainly implemented on animal models of transplantation. That is partially because MDSCs in mice are more precisely identified than MDSCs in humans. However, more experiments and preclinical trials on human volunteers or recipients are required to determine the safety and efficiency of MDSC-mediated treatment. With breakthroughs regarding specific surface markers of human MDSCs, we believe that immunotherapy based on MDSCs could benefit induction of immune tolerance in solid organ transplantation as well as hematopoietic stem cell transplantation in the near future.

## References

[1] Serafini P., Borrello I., Bronte V. (2006). Myeloid suppressor cells in cancer: recruitment, phenotype, properties, and mechanisms of immune suppression. *Seminars in Cancer Biology*.

[2] Gabrilovich D. I., Bronte V., Chen S.-H. (2007). The terminology issue for myeloid-derived suppressor cells. *Cancer Research*.

[3] Youn J.-I., Nagaraj S., Collazo M., Gabrilovich D. I. (2008). Subsets of myeloid-derived suppressor cells in tumor-bearing mice. *Journal of Immunology*.

[4] Bartmann C., Junker M., Segerer S. E., Hausler S. F., Krockenberger M., Kammerer U. (2016). CD33(+) /HLA-DR(neg) and CD33(+) /HLA-DR(+/-) cells: rare populations in the human decidua with characteristics of MDSC. *American Journal of Reproductive Immunology*.

[5] Nagaraj S., Gupta K., Pisarev V. (2007). Altered recognition of antigen is a mechanism of CD8+ T cell tolerance in cancer. *Nature Medicine*.

[6] Rodriguez P. C., Zea A. H., Culotta K. S., Zabaleta J., Ochoa J. B., Ochoa A. C. (2002). Regulation of T cell receptor CD3zeta chain expression by L-arginine. *The Journal of Biological Chemistry*.

[7] Rodriguez P. C., Quiceno D. G., Ochoa A. C. (2007). L-arginine availability regulates T-lymphocyte cell-cycle progression. *Blood*.

[8] Sawanobori Y., Ueha S., Kurachi M. (2008). Chemokine-mediated rapid turnover of myeloid-derived suppressor cells in tumor-bearing mice. *Blood*.

[9] Parker K. H., Sinha P., Horn L. A. (2014). HMGB1 enhances immune suppression by facilitating the differentiation and suppressive activity of myeloid-derived suppressor cells. *Cancer Research*.

[10] Egger G., Liang G., Aparicio A., Jones P. A. (2004). Epigenetics in human disease and prospects for epigenetic therapy. *Nature*.

[11] Vaknin I., Blinder L., Wang L. (2008). A common pathway mediated through Toll-like receptors leads to T- and natural killer-cell immunosuppression. *Blood*.

[12] Nausch N., Galani I. E., Schlecker E., Cerwenka A. (2008). Mononuclear myeloid-derived ‘suppressor’ cells express RAE-1 and activate natural killer cells. *Blood*.

[13] Li H. Q., Han Y. M., Guo Q. L., Zhang M. G., Cao X. T. (2009). Cancer-expanded myeloid-derived suppressor cells induce anergy of NK cells through membrane-bound TGF-*β*1. *The Journal of Immunology*.

[14] Hu C.-E., Gan J., Zhang R.-D., Cheng Y.-R., Huang G.-J. (2011). Up-regulated myeloid-derived suppressor cell contributes to hepatocellular carcinoma development by impairing dendritic cell function. *Scandinavian Journal of Gastroenterology*.

[15] Heim C. E., Vidlak D., Scherr T. D. (2014). Myeloid-derived suppressor cells contribute to staphylococcus aureus orthopedic biofilm infection. *Journal of Immunology*.

[16] Lui S. L., Chan K. W., Tsang R., Yung S., Lai K. N., Chan T. M. (2006). Effect of rapamycin on renal ischemia-reperfusion injury in mice. *Transplant International*.

[17] Poschke I., Mao Y., Adamson L., Salazar-Onfray F., Masucci G., Kiessling R. (2012). Myeloid-derived suppressor cells impair the quality of dendritic cell vaccines. *Cancer Immunology, Immunotherapy*.

[18] Greifenberg V., Ribechini E., Rößner S., Lutz M. B. (2009). Myeloid-derived suppressor cell activation by combined LPS and IFN-*γ* treatment impairs DC development. *European Journal of Immunology*.

[19] Rolinski J., Hus I. (2014). Breaking immunotolerance of tumors: a new perspective for dendritic cell therapy. *Journal of Immunotoxicology*.

[20] Beury D. W., Parker K. H., Nyandjo M., Sinha P., Carter K. A., Ostrand-Rosenberg S. (2014). Cross-talk among myeloid-derived suppressor cells, macrophages, and tumor cells impacts the inflammatory milieu of solid tumors. *Journal of Leukocyte Biology*.

[21] Ostrand-Rosenberg S., Sinha P., Beury D. W., Clements V. K. (2012). Cross-talk between myeloid-derived suppressor cells (MDSC), macrophages, and dendritic cells enhances tumor-induced immune suppression. *Seminars in Cancer Biology*.

[22] Mantovani A., Sica A., Allavena P., Garlanda C., Locati M. (2009). Tumor-associated macrophages and the related myeloid-derived suppressor cells as a paradigm of the diversity of macrophage activation. *Human Immunology*.

[23] Ostrand-Rosenberg S., Sinha P. (2009). Myeloid-derived suppressor cells: linking inflammation and cancer. *Journal of Immunology*.

[24] Bunt S. K., Clements V. K., Hanson E. M., Sinha P., Ostrand-Rosenberg S. (2009). Inflammation enhances myeloid-derived suppressor cell cross-talk by signaling through Toll-like receptor 4. *Journal of Leukocyte Biology*.

[25] Thakur A., Schalk D., Tomaszewski E. (2013). Microenvironment generated during EGFR targeted killing of pancreatic tumor cells by ATC inhibits myeloid-derived suppressor cells through COX2 and PGE_2_ dependent pathway. *Journal of Translational Medicine*.

[26] Shin J.-S., Ebersold M., Pypaert M., Delamarre L., Hartley A., Mellman I. (2006). Surface expression of MHC class II in dendritic cells is controlled by regulated ubiquitination. *Nature*.

[27] Thibodeau J., Bourgeois-Daigneault M.-C., Huppé G. (2008). Interleukin-10-induced MARCH1 mediates intracellular sequestration of MHC class II in monocytes. *European Journal of Immunology*.

[28] Marrero I., Ware R., Kumar V. (2015). Type II NKT cells in inflammation, autoimmunity, microbial immunity, and cancer. *Frontiers in Immunology*.

[29] Slauenwhite D., Johnston B. (2015). Regulation of NKT cell localization in homeostasis and infection. *Frontiers in Immunology*.

[30] Terabe M., Matsui S., Park J.-M. (2003). Transforming growth factor-*β* production and myeloid cells are an effector mechanism through which CD1d-restricted T cells block cytotoxic t lymphocyte-mediated tumor immunosurveillance: abrogation prevents tumor recurrence. *Journal of Experimental Medicine*.

[31] Hongo D., Tang X., Baker J., Engleman E. G., Strober S. (2014). Requirement for interactions of natural killer T cells and myeloid-derived suppressor cells for transplantation tolerance. *American Journal of Transplantation*.

[32] Safinia N., Scotta C., Vaikunthanathan T., Lechler R. I., Lombardi G. (2015). Regulatory T cells: serious contenders in the promise for immunological tolerance in transplantation. *Frontiers in Immunology*.

[33] Huang B., Pan P.-Y., Li Q. (2006). Gr-1+CD115+ immature myeloid suppressor cells mediate the development of tumor-induced T regulatory cells and T-cell anergy in tumor-bearing host. *Cancer Research*.

[34] Garcia M. R., Ledgerwood L., Yang Y. (2010). Monocytic suppressive cells mediate cardiovascular transplantation tolerance in mice. *The Journal of Clinical Investigation*.

[35] Kim Y. J., Chang S. Y., Ko H. J. (2015). Myeloid-derived suppressor cells in inflammatory bowel disease. *Intestinal Research*.

[36] Haile L. A., von Wasielewski R., Gamrekelashvili J. (2008). Myeloid-derived suppressor cells in inflammatory bowel disease: a new immunoregulatory pathway. *Gastroenterology*.

[37] Yang R., Cai Z., Zhang Y., Yutzy W. H., Roby K. F., Roden R. B. S. (2006). CD80 in immune suppression by mouse ovarian carcinoma-associated Gr-1^+^CD11b^+^ myeloid cells. *Cancer Research*.

[38] Dugast A.-S., Haudebourg T., Coulon F. (2008). Myeloid-derived suppressor cells accumulate in kidney allograft tolerance and specifically suppress effector T cell expansion. *The Journal of Immunology*.

[39] Dilek N., Poirier N., Usal C., Martinet B., Blancho G., Vanhove B. (2012). Control of transplant tolerance and intragraft regulatory T cell localization by Myeloid-derived suppressor cells and CCL5. *Journal of Immunology*.

[40] Meng F., Chen S., Guo X. (2014). Clinical significance of myeloid-derived suppressor cells in human renal transplantation with acute T cell-mediated rejection. *Inflammation*.

[41] Hock B. D., Mackenzie K. A., Cross N. B. (1460). Renal transplant recipients have elevated frequencies of circulating myeloid-derived suppressor cells. *Nephrology, Dialysis, Transplantation*.

[42] Zhang W., Liang S., Wu J., Horuzsko A. (2008). Human inhibitory receptor immunoglobulin-like transcript 2 amplifies CD11b+Gr1+ myeloid-derived suppressor cells that promote long-term survival of allografts. *Transplantation*.

[43] De Wilde V., Van Rompaey N., Hill M. (2009). Endotoxin-induced myeloid-derived suppressor cells inhibit alloimmune responses via heme oxygenase-1. *American Journal of Transplantation*.

[44] Adeegbe D., Serafini P., Bronte V., Zoso A., Ricordi C., Inverardi L. (2011). In vivo induction of myeloid suppressor cells and CD4^+^foxp3^+^ T regulatory cells prolongs skin allograft survival in mice. *Cell Transplantation*.

[45] Marigo I., Bosio E., Solito S. (2010). Tumor-induced tolerance and immune suppression depend on the C/EBP*β* transcription factor. *Immunity*.

[46] Chou H.-S., Hsieh C.-C., Charles R. (2012). Myeloid-derived suppressor cells protect islet transplants by B7-H1 mediated enhancement of T regulatory cells. *Transplantation*.

[47] Arakawa Y., Qin J., Chou H.-S. (2014). Cotransplantation with myeloid-derived suppressor cells protects cell transplants: a crucial role of inducible nitric oxide synthase. *Transplantation*.

[48] Bryant J., Lerret N. M., Wang J.-J. (2014). Preemptive donor apoptotic cell infusions induce IFN-*γ*-producing myeloid-derived suppressor cells for cardiac allograft protection. *The Journal of Immunology*.

[49] Nakamura T., Nakao T., Yoshimura N., Ashihara E. (2015). Rapamycin prolongs cardiac allograft survival in a mouse model by inducing myeloid-derived suppressor cells. *American Journal of Transplantation*.

[50] Zhou Z., French D. L., Ma G. (2010). Development and function of myeloid-derived suppressor cells generated from mouse embryonic and hematopoietic stem cells. *Stem Cells*.

[51] Highfill S. L., Rodriguez P. C., Zhou Q. (2010). Bone marrow myeloid-derived suppressor cells (MDSCs) inhibit graft-versus-host disease (GVHD) via an arginase-1-dependent mechanism that is up-regulated by interleukin-13. *Blood*.

[52] Wang D., Yu Y., Haarberg K. (2013). Dynamic change and impact of myeloid-derived suppressor cells in allogeneic bone marrow transplantation in mice. *Biology of Blood and Marrow Transplantation*.

